# Cyclin‐dependent kinase subunit2 (CKS2) promotes malignant phenotypes and epithelial‐mesenchymal transition‐like process in glioma by activating TGFβ/SMAD signaling

**DOI:** 10.1002/cam4.5381

**Published:** 2022-10-25

**Authors:** Fan Feng, Zongqing Zhao, Xuechang Cai, Xueyuan Heng, Ximeng Ma

**Affiliations:** ^1^ Institute of Clinical Medicine College, Guangzhou University of Chinese Medicine Guangzhou China; ^2^ Institute of Brain Science and Brain‐Like Intelligence, Linyi People's Hospital Linyi China; ^3^ Department of Neurosurgery Linyi People's Hospital Linyi China; ^4^ Department of Neurosurgery Qingdao Huangdao District Central Hospital Qingdao China

**Keywords:** apoptosis, CKS2, EMT, glioma, TGFβ/SMAD signaling

## Abstract

**Background:**

Gliomas are a group of primary intracranial tumors with high morbidity and mortality. The previous researches indicated a crucial role of CKS2 (cyclin‐dependent kinases regulatory subunit 2) in hepatocellular carcinoma and breast cancer; however, little is known about the molecular mechanism of CKS2 in the tumorigenesis and epithelial‐mesenchymal transition‐like (EMT) process in glioma.

**Methods:**

Datasets for bioinformatics analysis were obtained from the GEO, TCGA and CGGA databases. qRT‐PCR, western blotting (WB), and immunohistochemistry (IHC) assays were used to investigate the expression patterns of CKS2 among glioma and brain tissues. Glioma cells were transfected with small interfering RNA/overexpression plasmid against CKS2, then clone formation assay, CCK‐8, wound healing, Transwell assay, and flow cytometry were performed to detect changes in cell viability, invasiveness, and the apoptosis rate. Markers of cell invasion, apoptosis, EMT and TGFβ/SMAD signaling were evaluated by WB and immunofluorescence (IF) assays.

**Results:**

We found that CKS2 overexpression correlates with poor prognosis in human glioma and knockdown of CKS2 could inhibit cell proliferation, migration, invasion, and induced apoptosis in glioma cells. Besides, we also found that knockdown of CKS2 could reverse the EMT process via modulating EMT‐related molecules. Glioma cells with overexpression of CKS2 were constructed to confirmed the fact that CKS2 induced nucleocytoplasmic translocation of SMAD2/3 and activated TGFβ/SMAD pathway, then upregulated its downstream targets expression, while inhibition of TGFβ/SMAD (by TGFβ inhibitor LY2157299 or SMAD4 siRNA) could reverse the tumor‐promoting effects and malignant phenotype caused by CKS2 overexpression.

**Conclusions:**

We identified CKS2 as a critical contributor to the gliomagenesis, which might provide a novel therapeutic target for inhibiting the spread and infiltration of glioma.

## Graphical abstract

Cyclin‐dependent kinase subunit2 (CKS2) is small highly conserves cyclin‐dependent kinase (CDK)‐interacting proteins (10KDa), which located in chromosome 9q22. CKS2, as a crucial member of CKS family, plays an important role in the process of gliomagenesis. DNA‐binding dynamics of CKS2 showed a cell cycle‐dependent manner; CKS2 knockdown inhibited cell proliferation and induced apoptosis. We also found CKS2 overexpression could induced the nucleocytoplasmic translocation of SMAD2/3 and upregulated P‐TGFßR1, P‐samd2, P‐smad3, and P‐smad7. In addition, CKS2‐mediated activation of TGFß/SMAD signaling pathway could induce aggressiveness and malignant phenotype (epithelial‐mesenchymal transition) in glioma.

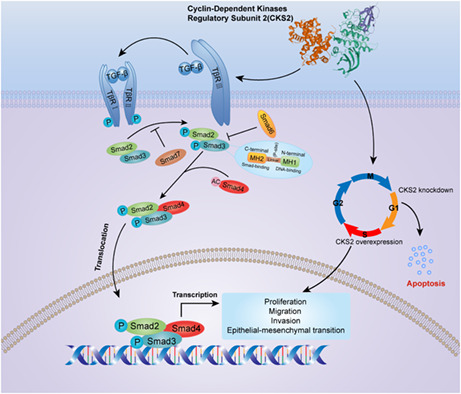

## 1 INTRODUCTION

Glioma, especially glioblastoma multiforme (GBM), is the most common and lethal human primary brain tumor, which characterized by rapid progress, poor prognosis, and resistance to chemotherapy and radiotherapy.^1^, ^2^, ^3^ Despite advance in the surgical intervention, the median survival time of patients with glioblastoma is approximately 14.6 months .^4^ Hence, better recognition of the molecular mechanisms involved in gliomagenesis is imperative to highlight potential targets and novel strategies for treating glioma.

Cyclin‐dependent kinase subunit2 (CKS2) is small highly conserves cyclin‐dependent kinase (CDK)‐interacting proteins (10KDa), which located in chromosome 9q22.^5^ CKS2, as a crucial member of CKS family, plays an important role in the formation of human embryo, cell cycle regulation and the process of somatic cell division.^6^, ^7^, ^8^ These evidences suggested that CKS2 may exert a functional role in the process of tumorigenesis.^9^, ^10^ Indeed, accumulating findings showed that CKS2 expression was abnormally elevated in several tumors and acted as a tumor‐promotive biomarker. For instance, Yu et al reported that overexpression of CKS2 was strongly associated with poor prognosis via analyzing clinical data from 183 patients with colorectal cancer (CRC); besides, knockdown of CKS2 could promote cell apoptosis and inhibit the tumorigenesis through modulating claudin1 expression.^11^ In AMC‐HN8 cells, CKS2, as a direct target of miR‐26a, induced cell migration and invasion, suggesting that CKS2 could be a novel therapeutic target for laryngeal squamous cell carcinoma (LSCC).^12^ Importantly, an oligonucleotide microarray analysis also reported that CKS2mRNA was highly and frequently amplified in primary glioblastomas.^13^, ^14^ These evidences above illustrated that CKS2 might be a new therapeutic target for gliomagenesis; however, the molecular mechanism has not yet been investigated.

Epithelial‐mesenchymal transition (EMT) is a complex reversible cell remodeling process involving high phenotypic plasticity during tumor progression and embryonic development. EMT is thought to be activated in tumor metastasis, linked to lose their cell adhesion, cell polarity and gain invasion properties.^15^ Nowadays, EMT‐like process is widely accepted in central nervous system (CNS) tumors.^16^ Although it is low rate for glioma patients to present tumor metastasis, Awan et al reported that circulating tumor cells were detected in 20% GBM patients' blood.^17^ Besides, Nesvick also demonstrated that EMT‐promoting transcription factors (EMT‐IFs) are involved in the initiation and progression of primary II ~ III gliomas.^18^ Hence, EMT plays an important role in maintaining malignant feature of glioma and promoting spread. The multi‐step, complex progress of EMT contained multiple regulation molecular mechanism, such as ERK–MAPK, Rac1/RhoA, Wnt/β‐catenin pathway, and so forth.^19^, ^20^, ^21^ Recently, Jiang et al supported the notion that TGF‐β/Smad signaling pathway was involved in the EMT‐like process during the gliomagenesis.^22^

Transforming growth factor β (TGF‐β) is a disulfide‐bonded dimer protein containing three isoforms, TGF‐β1, TGF‐β2, and TGF‐β3, among which TGF‐β1 is the predominant isoform. In previous studies, TGF‐β acted as a multifunctional cytokine controlling angiogenesis, cell differentiation, immune response, and proliferation.^23^, ^24^ Current studies have found that TGF‐β had a dual role in tumor pathology, which was depended on the type of cancer. Solvated metal atom dispersed (SMAD), as a classical TGF‐β dependent protein, contains spherical MH domains and a cohesive variable zone. When binding to TGF‐β, the activated SMADs transfer to the nucleus to regulated downstream targets transcription and expression.^25^ Some scholars have found that activated TGFβ/SMAD pathway was involved in the tumorigenesis via regulation a variety of pathophysiological process, among which epithelial‐mesenchymal transition (EMT) is widely studied.^26^, ^27^ For example, Tian et al demonstrated that the Fork‐head activin signal transducer 1 (FOXH1) promotes migration of 769‐P and ACHN Renal carcinoma cells by regulating the expression of EMT markers (Claudin1, Snail, N‐cadherin, E‐cadherin) and activating TGFβ/SMAD signaling pathway.

At present study, we first reported that expression of CKS2 was aberrantly elevated in glioma with surrounding spread and infiltration, and CKS2 overexpression was related to proliferation, invasion, and migration of glioma cells and shorter survival time of glioma patients. Furthermore, CKS2 promoted the EMT‐like process through TGFβ/SMAD signaling pathway in glioma, meanwhile, inhibition of TGFβ/SMAD could reverse the malignant phenotype caused by CKS2, which implied that CKS2 may serve as a potential target for the treatment of glioma.

## 2 MATERIALS AND METHODS

### 2.1 Tissue sample collection and ethics statement

The present study was carried out in Linyi People's Hospital. A total of 70 glioma patients who were diagnosed, surgically resected, and pathologically confirmed were enrolled in our study. Parts of fresh glioma tumor tissues were immediately transferred to liquid nitrogen and stored at −80°C for subsequent Western blotting (WB) and qRT‐PCR assays. The remaining tissues were fixed in 4% paraformaldehyde (PFA) for immunohistochemical staining. None of glioma patients had received any treatment before tumor resection procedures, such as radiotherapy or chemotherapy. This study was approved by the ethnics committee of Linyi People's Hospital and in accordance with the Helsinki Declaration. All patients had signed the informed contents.

### 2.2 Microarray data preparation

Microarray RNA‐seq data of glioma patients were downloaded from the GEO database (https://www.ncbi.nlm.nih.gov/geo/) under the accession number GSE7696, GSE54004, GSE4290, GSE16011, and GSE67089. The GPL570 (Affymetrix Gene Chip Human Genome U133 Plus 2.0 Array), GPL18281 (Illumina HumanHT‐12 WG‐DASL V4.0 R2 expression beadchip), and GPL13667 (Affymetrix Human Genome U219 Array) platforms were used. The clinical data and related RNA‐seq data from patients with glioma were obtained from CGGA (mRNA_array_301; http://www.cgga.org.cn/) and TCGA databases (https://xenabrowser.net/datapages/).

### 2.3 Quantitative real‐time polymerase chain reaction

TRIZOL method (Takara) was adopted for extracting the total RNA from tissue and cells according to the protocol. The sample RNA was reserved into cDNA by using a cDNA Synthesis Kit (Takara). Quantitative real‐time polymerase chain reaction (qRT‐PCR) reactions were performed by using the SYBR Green Fast Real‐Time PCR system. The primers synthesized by Sangon Biotech. CKS2‐forward: 5′‐AG AGGAGTGGAGGAGACTTGG‐3′; CKS2‐revise: 5′‐TGTGG TTCTGGCTCATGAAT‐3′; GAPDH‐forward: 5′‐TGCACCA CCAACTGCTTAGC‐3′; GAPDH‐revise: 5′‐GGCA TGCAC TGTGGTCATGAG‐3′. The experiment was repeated three times.

### 2.4 Cell culture

Human glioma cell lines A172, U251, TJ905, LN229, PT2, and SF295 and human astrocyte cell line HA 1800 were purchased from the Cell Bank of Shanghai institutes for Biological Science. All cells were cultured in Dulbecco's modified Eagle's media (DEME; Gibco Life Technologics) supplemented with 10% fetal bovine serum (FBS; Hyclone). The cells were incubated at 37°C with 5% CO2.

### 2.5 Reagents and antibodies

CKS2 rabbit mAb (AF0616; WB 1:2000; IHC 1:150; IF 1:300); SAMD2/3 rabbit mAb (AF6367; WB 1:1500); Phospho‐SAMD2 rabbit mAb (AF3450; WB 1:2500); Phospho‐SAMD3 rabbit mAb (AF3362; WB 1:2500); Phospho‐SAMD7 rabbit mAb (AF3827; WB 1:2500); E‐cadherin rabbit mAb (No.20874‐1‐AP; WB 1:4000; IF 1:200); N‐cadherin rabbit mAb (No.22018‐1‐AP; WB 1:3000; IF 1:300); Snail rabbit mAb (No.26183‐1‐AP; WB 1:800); Vimentin rabbit mAb (No.10366‐1‐AP; WB 1:3000); BAX rabbit mAb (50599‐2‐Ig; WB 1:3000); BCL‐2 rabbit mAb (12789‐1‐AP; WB 1:3000); Caspase3 rabbit mAb (AF6311; WB 1:2500); Cleaved‐Caspase3 rabbit mAb (AF7022; WB 1:2500); EF‐1α rabbit mAb (DF9493; WB 1:2500); GAPDH mouse mAb (No.60004‐1‐Ig; WB 1:3000). Antibodies describe above were purchased from Proteintech and Affinity Biosciences. TGFβ/SMAD pathway inhibitor (LY2157299) was purchased from Selleck.

### 2.6 Western blot analysis

Cells were lysed in pre‐cold radio immunoprecipitation (RIPA) buffer; then, protein samples were extracted and quantified by bicinchoninic acid (BCA) protein assay kit. Protein mixed with loading buffer was separated by SDS‐PAGE, then, the transferred‐ PVDF membrance was blocked by 5% skim milk at 4°C for 1 hr. Primary antibody was incubated overnight and washed three time with PBST. The PVDF membrane was again washed three times after being incubated with HPR‐labeled secondary antibody. The blots were visualized by a Western blot detection system. GAPDH or EF1‐α was used as the endogenous reference according to details. All assays were independently performed in triplicate.

### 2.7 Transfection procedure

To overexpress CKS2, the coding sequence was obtained from the NCBI and was synthesized to facilitate cloning. The synthesized sequence was ligated and subcloned into pBABE lentivirus vector, and an empty vector was used as the control group. Two siRNA targeting CKS2 and SMAD4 were used in the study, including CKS2 siRNA (RNAi#1, 5′‐GCUGGGUUCAUUACAUGA UdTdT‐3′ and RNAi#2, 5′‐CAGAACCACAUAUUCUUCUdTdT‐3′) and SMAD4 siRNA (RNAi#1, 5′‐GCATGTTAAGGAAACTCAGCC‐3′ and RNAi#2, 5′‐GCAGCGCCTCATCAAGAAAGT‐3′). Negative siRNA and empty vector were used as the control group. The siRNAs and lentiviral particles were transfected into cells using Lipofectamine 3000 (Invitrogen) according to the manufacturer's protocol. Puromycin was used to screen the stable cells, which were collected for subsequent experiments.

### 2.8 CCK‐8 and colony formation assay

For cell counting kit‐8 assay, CCK‐8 reagent was added into each well of 96‐well plates; then, absorbance of each well was assessed at 450 nm under automatic microplate reader. Cell viability was also assessed using the cell counting kit 8 (CCK‐8, Beyotime) method at 0, 24, 48, and 72 h. For colony formation assay, cells were seeded into 6‐well plates(1 × 10^3^/well) and incubated for 14 days until visible clones appeared; then, cells were fixed under 4% methyl alcohol for 20mins and stained using 5% crystal violet for 15 mins. Finally, clones were counted under a microscope.

### 2.9 Wound healing assay and transwell assay

For wound healing assay, cells were seeded in 6‐well plates and wounds were made with yellow pipette tips; then, the cell monolayers were washed twice with PBS. Images of the width of wound were photographed at 0 h, 36 h, and 72 h later. For transwell assay, cells were added in the upper chamber coated with a Matrigel basement membrane matrix (BD, Bioscience); besides, the low chamber was supplemented with 10% fetal bovine serum (FBS). Finally, glioma cells that passed through the membrane were stained with crystal violet and visualized under a microscope.

### 2.10 Immunohistochemistry staining

All tissue section (3 μm thick) dried at 70°C for 40 mins; then, endogenous peroxidase activity was quenched with 3% hydrogen peroxide for 10 mins and blocked with normal goat serum. Serial tissue sections were incubated with horseradish peroxidase‐conjugated secondary antibody (Zhongshan Golden Bridge Biotechnology) for 30 mins at 4°C. The specimens were visualized with DAB. We adopted a previously reported method to assess the expression of CKS2 as follows: the percentage of positive cell scores: [score 0, no staining; score 1, <10%; score 2, 10%–30%; and score 3, >30%]. The staining intensity score: [score 0, negative; score 1, weak intensity; score 2, moderate intensity; and score3 strong intensity]. Sum score <4 means negative, whereas sum scores ≥ 4 was considered as positive.^11^

### 2.11 Immunofluorescence analysis

Glioma cells grown on coverslips in 6‐well plate overnight were washed with PBS and fixed with 4% paraformaldehyde for 15 mins. Coverslips were permeabilized by 0.1% Triton X‐100 for 10 mins and blocked with 5% BSA at 4°C for 30 mins. They were incubated with primary antibody, then washed with PBS, and incubated with secondary antibody. The nuclei were stained with DAPI at 4°C for 10 mins. Finally, slides were observed under a fluorescence microscope (Olympus).

### 2.12 Cell apoptosis assay

Collected glioma cells were washed by PBS and then fixed in 70% ice‐cold ethanol overnight at 4°C. The cells were resuspended in PBS containing RNAase and propidium iodide (PI) at room temperature for 30 mins after washed twice with PBS. Apoptosis was analyzed using the PE Annexin V apoptosis detection kit (BD pharmingen) according to the manufacturer's protocol. U251 and LN229 cells transfected with CKS2 overexpression plasmid, vector, CKS2‐siRNA, and negative‐siRNA were cultured for 72 h and digested by 0.1%trysin. Then, the glioma cells were resuspended at a concentration of 1 × 10^6^ cells/ml in Binding Buffer, and then, 5 μL of PI and 10 μL of Annexin V‐FITC were added. After 15 mins incubation at room temperature in the dark, 400 μL of 1 × Binding Buffer was added to the cell suspension. Subsequently, the apoptosis assay was performed by flow cytometry (BD, Bioscience). All assays were independently performed in triplicate.

### 2.13 Bioinformatics analysis

Gene ontology (GO) and Kyoto Encyclopedia of Genes and Genomes (KEGG) enrichment analysis were performed to analyze the genes associated with CKS2. Enriched ontological terms and pathways with *p* < 0.05 were selected and presented in a barplot using the R package “clusterProfiler,” “ggstatsplot,” “stringr,” “dplyr,” and “tidyr” (http://www.bioconductor.org/pack/).

Gene set enrichment analysis (GSEA) was performed to generate an ordered list of all genes related to the level of CKS2. The expression of CKS2 was used as a phenotype label. The normalized enrichment score (NES) and false discovery rate (FDR) were performed to sort enriched biological pathways in each phenotype. Other R packages, “ggpubr,” “ggplot2,” and “limma” were also applied to visualize the results of bioinformatics analysis.

### 2.14 Statistical analysis

Statistical analysis was performed using Origin2018 and Sigmaplot version 14.0 for Windows. Data were expressed as the Means ± SD. Student's *t*‐test was used to analyze differences between two groups. Comparisons between more than three groups were determined using one‐way ANOVA analysis of variance followed by the Turkey post‐hoc test. The chi‐square test examines the relationship between CKS2 and clinicopathological characteristics. Kaplan–Merier survival analysis was used to analyze overall survival (OS) time. A value of *p* < 0.05 was considered statistically significant.

## 3 RESULTS

### 3.1 High expression of CKS2mRNA in human glioma correlates with poor prognosis

**FIGURE 1:**
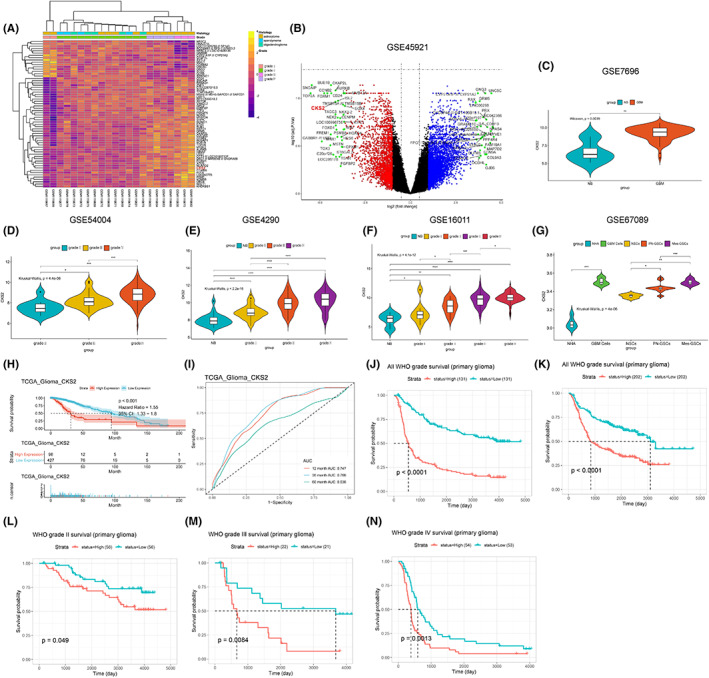
CKS2 expression patterns in glioma. (A, B) Heatmap and volcano plots of differentially expressed genes (DEGs) in glioma based on data from GSE45921. (C–F) Based on analysis of RNA‐seq data from GSE7696, GSE54004, GSE4290, and GSE16011, CKS2mRNA was significantly increased in high‐grade glioma. (G) Compared with the normal human astrocyte (NHA) and neuro stem cells (NSCs), CKS2 presents aberrant overexpression in glioblastoma (GBM) cells and glioma stem cells (GSCs). (H–I) Kaplan–Meier curve comparing overall glioma patients according to the level of CKS2 in TCGA database, the ROC curve showed that CKS2 had a large area under the curve (AUC) in patients with diagnose time 12 months, 30 months, and 60 months. (Respectively, AUC were 74.7%, 76.6%, and 63.6%) (J–N) K‐M survival curves comparing the high and low expression of CKS2 in glioma patients with different grade, data from CGGA database.

Firstly, CKS2mRNA was systematically screened as dysregulated gene in gliomagenesis through microarray gene expression database with different WHO grade (GEO database: GSE 45921, Figure. 1A, B). To investigate the clinical significance of CKS2 in human glioma, we initially analyzed the RNA‐seq data using the GSE7696, GSE54004, GSE4290, and GSE16011 datasets. The statistical results of the four datasets indicated that the level of CKS2mRNA was significantly upregulated in glioma compared to normal brain tissues (Figure. 1C–F). The astrocyte and glioma cells were differentiated from neuro stem cells (NSCs) and glioma stem cells (GSCs), respectively. NSCs, as the most active cells in central nervous system (CNS), are in a state of continuous proliferation and division and are prone to mutation; hence, mutated NSCs may transform into GSCs. In the GSE67089 microarray, CKS2mRNA was significantly overexpressed in GSCs and GBM cells compared with NHA and NSCs, which suggested that CKS2 might play a crucial role in gliomagenesis (Figure. 1G). The expression pattern of CKS2mRNA in glioma was also confirmed in CGGA database. To evaluate the association between CKS2mRNA expression and clinical outcomes of patients with glioma, we performed Kaplan–Meier (KM) survival analysis from TCGA and CGGA microarray database. The results showed that the overall survival (OS) of glioma patients with high CKS2mRNA expression was shorter (Figure. 1H–K). Considering the biological heterogeneity between different WHO tumor grade in glioma, we further explore the prognostic value of CKS2mRNA in patients with various WHO grade from CGGA database. After dividing patients into two equal groups according to the level of CKS2mRNA, we also found that patients stratified by a media cutoff of CKS2mRNA level with lower CKS2mRNA expression had longer OS than those with higher levels of CKS2mRNA (Figure. 1L–N). Those evidences mentioned above demonstrated that CKS2 was a negative prognostic factor in glioma patients.

### 3.2 CKS2 protein overexpression correlates with poor prognosis in human glioma

**FIGURE 2:**
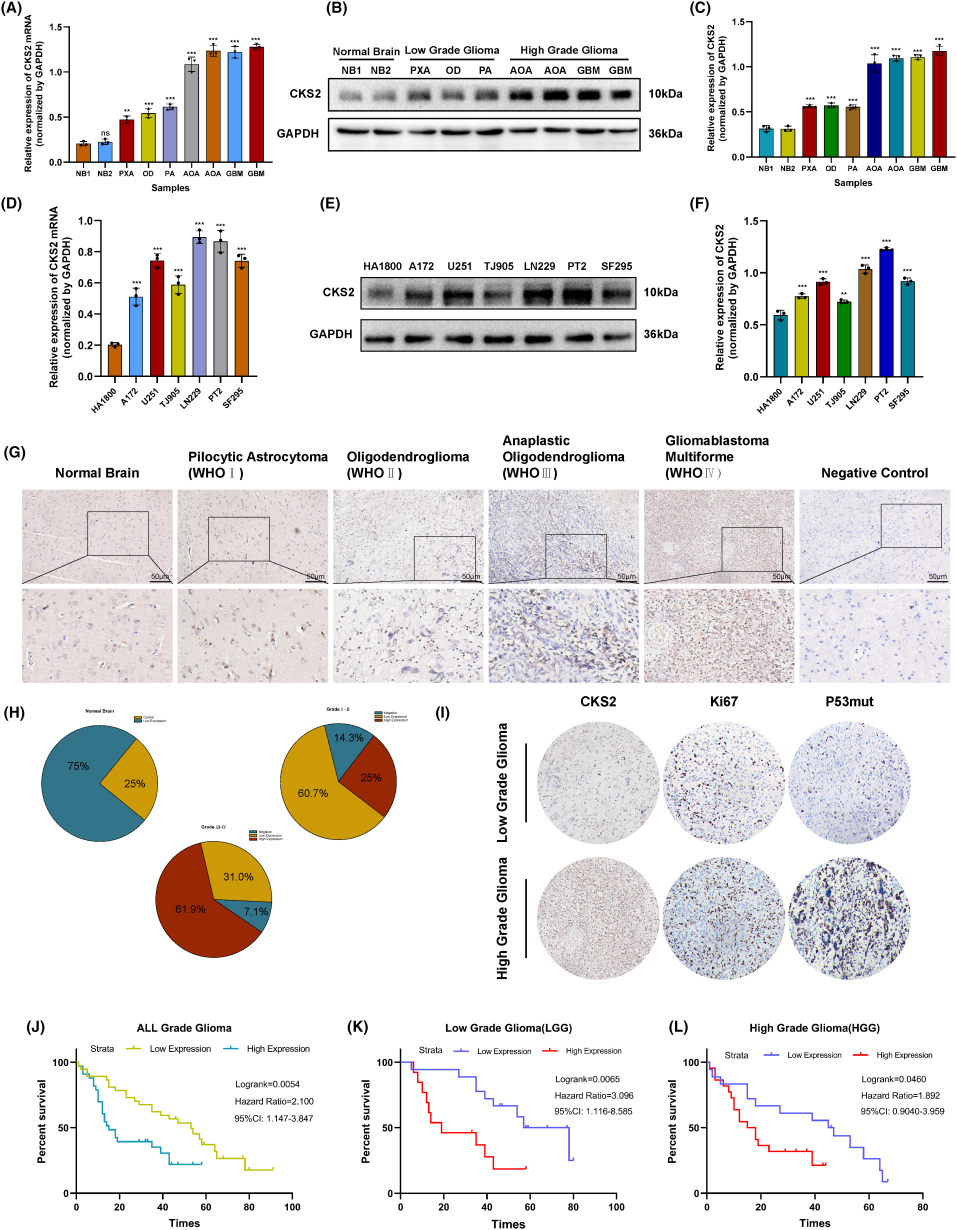
High CKS2 expression predicted poor prognosis in glioma patients. (A–C) qRT‐PCR and Western blot analysis of CKS2 in glioma tissues and non‐tumor tissues collected from Linyi People's Hospital. (D–F) mRNA and protein level of CKS2 in glioma cell lines and normal astrocyte cell lines. (G, H) The expression of CKS2 in normal brain tissues and 70 glioma tissues, measured by immunohistochemistry assay. IHC‐stain sections, scale bar, 50 μm. (I) IHC analysis of CKS2 and several clinicopathological parameters (Ki67 and P53mut). (J, K) Kaplan–Meier analysis for overall survival time in patients with glioma (Linyi People's Hospital, *n* = 70) based on CKS2 expression pattern.

To further validate the above conclusion, we detected the level of CKS2mRNA and protein in clinical sample and glioma cell lines. Consistent with the results of public database (GEO, CGGA), both CKS2mRNA and protein were aberrantly elevated in glioma tissues in tandem with increased tumor grade. Consistent with the findings in the RNA‐seq datasets, the results of qRT‐PCR and WB assays showed that CKS2 mRNA and proteins were significantly increased in glioma tissues compared to normal brain tissues (Figure. 2A–C). Besides, the expression of not only CKS2mRNA but also protein was markedly upregulated in glioma cell lines (U251, A172, PT2, SF 295, LN229, and TJ905) compared with normal human astrocyte (NHA) (Figure. 2D–F). Then, we further reviewed the clinical characteristics of 80 patients with glioma and explore the association between CKS2 expression and the clinicopathological features via immunohistochemistry assay (Table S1). Findings represented weak/negative staining for CKS2 in normal brain sample, moderate to strong cytoplasmic staining for CKS2 in low‐grade glioma (such as Pilocytic astrocytoma, Pleomorphic xanthoastrocytoma, and Oligodendroglioma) and high‐grade glioma (such as Anaplastic Oligodendroglioma and Glioblastoma Multiforme) tissues (Figure. 2G, H). Moreover, overexpression of CKS2 protein was significantly correlated with WHO grade as well as several malignant proliferation indicators such as Ki67 and P53mut (Figure. 2I, Table 1). Among 80 patients with glioma in this study, the eight tissues of GBM patients that represented multifocal feature markedly expressed CKS2 in either the core or margin of tumor. Interestingly, the other GBM patients in our study had circumscribed margins in MRI that often exhibited relatively lower CKS2 expression compared with the GBM tissues characterized by the severe invasion of adjacent brain (Figure. 2G). Follow‐up data of our research were collected from all 80 patients; Kaplan–Meier survival curve indicated that the CKS2 overexpression was correlated with significantly shorter OS in patients, whether at WHOI~II(LGG) or at WHO grade III ~ IV(HGG) (Figure. 2J–L). Finally, by utilizing the Logistics/Cox regression model, we further computed both logistics odds ratio and univariate/multivariate hazard ratios for different variables of 696 glioma patients from TCGA database. Logistics regression analysis found that high expression of CKS2 and higher WHO grade (odds ratio [OR], 13.243 [8.761–20.541 CI]; *p* < 0.001), IDH1 status (OR, 0.097 [0.065–0.141 CI]; *p* < 0.001), and 1p/19q codeletion (OR, 2.211 [1.550–3.178 CI]; *p* < 0.001) were significantly correlated. Univariate and multivariate analyses reported that factors such as CKS2 expression level and WHO grade were significantly correlated with the prognosis of patients with glioma (Table S2; Tables 2 and 3). Taken together, CKS2 protein upregulation contributed to gliomagenesis and correlated with poor prognosis of glioma.

**TABLE 1:** CKS2 expression associated with several clinicopathological parameters

Clinicopathological	CKS2 expression			
Parameter, Value	*N*	Low expression	High expression	*p* Value
Gender				
Male	32(45.7)	16(50.0)	16(50.0)	0.509
Female	38(54.3)	16(42.1)	22(57.9)	
Age				
<44	23(32.9)	9(39.1)	14(60.9)	0.439
≥44	47(67.1)	23(48.9)	24(51.1)	
Tumor diameter				
<4 cm	27(38.6)	11(40.7)	16(59.3)	0.508
≥4 cm	43(61.4)	21(48.8)	22(51.2)	
Tumor location				
Frontal	27(38.6)	15(55.6)	12(44.4)	0.365
Temporal	25(35.7)	9(36.0)	16(64.0)	
Other	18(25.7)	8(44.4)	10(55.6)	
KPS				
<80	23(32.9)	13(56.5)	10(43.5)	0.204
≥80	47(67.1)	19(40.4)	28(59.6)	
WHO grades				
I–II	28(40.0)	19(67.9)	9(32.1)	0.002*
III–IV	42(60.0)	13(31.0)	29(69.0)	
MGMT expression				
Low	41(58.6)	21(51.2)	9(36.0)	0.272
High	29(41.4)	11(37.9)	29(64.4)	
P53mut expression				
Low	25(35.7)	16(64.0)	9(36.0)	0.022*
High	45(64.3)	16(57.8)	29(64.4)	
Ki‐67 expression				
Low	27(38.6)	17(63.0)	10(37.0)	0.022*
High	43(61.4)	15(34.9)	28(65.1)	

* *p* < 0.05

* *p* < 0.01

* *p* < 0.001.

**TABLE 2:** Logistics regression analysis of OS in the patients with glioma (TCGA)

Characteristics	Total (*N*)	Odds Ratio (OR)	*p* Value
WHO grade (G3&G4 vs. G2)	635	13.243 (8.761–20.541)	<0.001*
1p/19q codeletion (non‐codel vs. codel)	689	2.211 (1.550–3.178)	<0.001*
Primary therapy outcome (PR&CR vs. PD&SD)	462	0.722 (0.488–1.065)	0.102
Gender (Male vs. Female)	696	1.024 (0.758–1.383)	0.878
IDH status (Mut vs. WT)	686	0.097 (0.065–0.141)	<0.001*

Abbreviations: CR, complete response; OS, overall survival; PD, progressive disease; PR, partial response; SD, stable disease.

* *p* < 0.05

* *p* < 0.01

* *p* < 0.001.

**TABLE 3:** Univariate/multivariate COX proportional hazard analysis of OS in the glioma patients (TCGA)

Characteristics	Total (N)	Univariate analysis		Multivariate analysis	
		Hazard ratio (95% CI)	*p* value	Hazard ratio (95% CI)	*p* value
WHO grade	634				
G2	223	Reference			
G3	243	2.999 (2.007–4.480)	<0.001*	2.032 (1.145–3.606)	0.015*
G4	168	18.615 (12.460–27.812)	<0.001*	4.590 (1.172–17.976)	0.029*
1p/19q codeletion	688				
Codel	170	Reference			
Non‐codel	518	4.428 (2.885–6.799)	<0.001*	1.698 (0.852–3.385)	0.133
Primary therapy outcome	314				
PR	64	Reference			
CR	138	0.767 (0.266–2.213)	0.623	0.834 (0.247–2.813)	0.770
PD	112	6.176 (2.684–14.210)	<0.001*	5.194 (1.862–14.486)	0.002*
Age	695				
≤60	552	Reference			
>60	143	4.668 (3.598–6.056)	<0.001*	4.322 (2.346–7.963)	<0.001*
IDH status	685				
WT	246	Reference			
Mut	439	0.117 (0.090–0.152)	<0.001*	0.592 (0.320–1.096)	0.096
Gender	695				
Female	297	Reference			
Male	398	1.262 (0.988–1.610)	0.062	2.048 (1.233–3.404)	0.055
CKS2	695				
Low	348	Reference			
High	347	4.398 (3.332–5.804)	<0.001*	1.154 (0.676–1.971)	0.006*

Abbreviations: CI, confidence interval; CR, complete response; OS, overall survival; PD, progressive disease; PR, partial response; SD, stable disease.

* *p* < 0.05

* *p* < 0.01

* *p* < 0.001.

### 3.3 Knockdown of CKS2 inhibits proliferation and induces apoptosis in glioma cells

**FIGURE 3:**
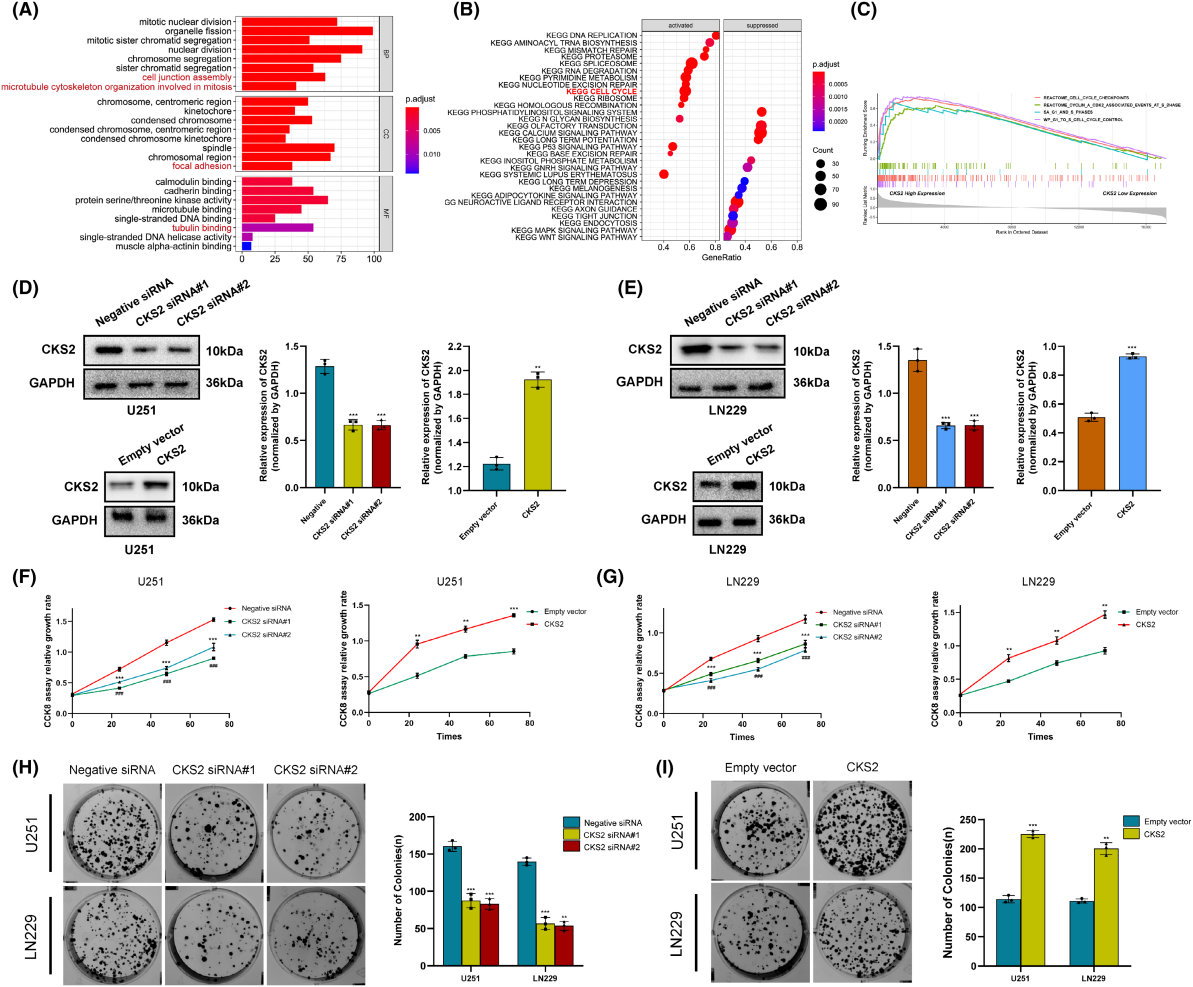
Knockdown of CKS2 inhibited cell proliferation in glioma. (A) Biological process, cell components, and molecular function enrichment analysis of CKS2. (B, C) Gene set enrichment analysis (GSEA) pathways enriched by CKS2. (D, E) The interference effects of CKS2siRNA and overexpression plasmid in U251 and LN229 cell lines. (F, G) Proliferation in U251 and LN229 glioma cells was measured by the CCK‐8 assay. (H, I) Colony formation analysis of LN229 and U251 cells treated with overexpression plasmid or CKS2siRNA after 14 days.

To further explore the roles of CKS2 in tumorigenesis of glioma, we predicted its potential functions via synergistic genes enrichment analysis and gene set enrichment analysis (GSEA). The function of CKS2 was then explored in‐depth via GO functions enrichment analysis; results demonstrated that CKS2 was involved in proliferation and migration (cell junction, focal adhesion, and microtubule cytoskeleton organization). Moreover, CKS2 gene expression also positively correlated with cell cycle and G1/S cell cycle control in GSEA database (Figure.3A–C).

**FIGURE 4:**
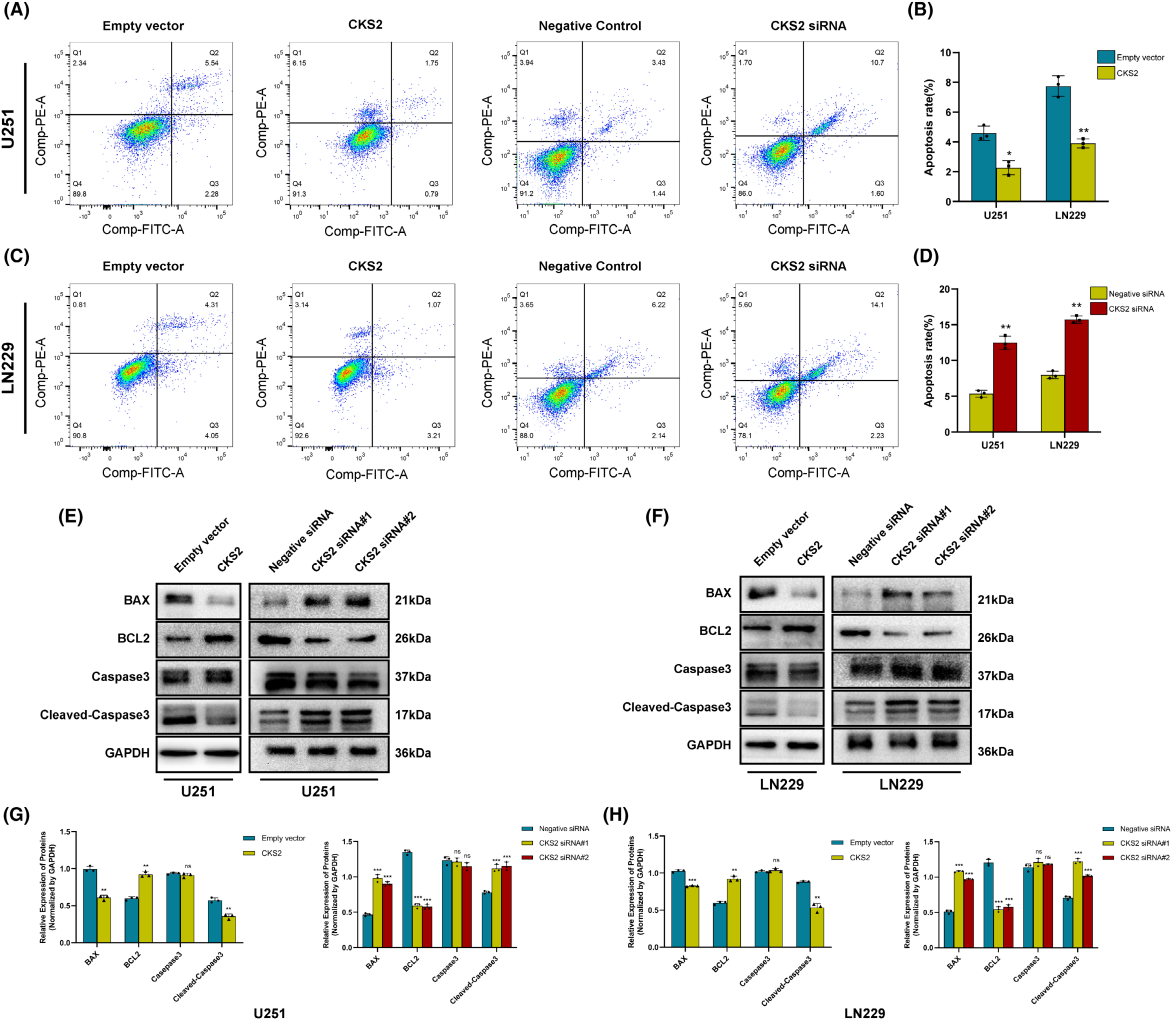
CKS2 promoted apoptosis in U251 and LN229 cells. (A‐D) U251 and LN229 cells were treated with negative siRNA, CKS2‐siRNA, empty vector, and CKS2 overexpression plasmid respectively, and then assayed using Flow Jo V10 Analyzer according to the manufacturer's protocol. (E, H) Western blot was used for the analysis of the level of apoptosis‐related protein (BAX, BCL2, Cleaved‐caspase3, and total caspase3) expression in overexpression plasmid and siRNA transfected U251 and LN229 cells.

Therefore, we investigate the effect of CKS2 on proliferation of glioma cells. CCK8 assay was performed and revealed that CKS2 overexpression promoted U251 and LN229 cell proliferation (Figure. 3D–G). In colony formation assay, overexpressing endogenous CKS2 significantly increased the viability of the glioma cells, which formed bigger and more clones. Conversely, knocking down CKS2 in U251 and LN229 cells greatly inhibited their proliferation (*p* < 0.05, Figure. 3H, I). To determine whether CKS2 is involved in apoptosis, several anti‐apoptotic and pro‐apoptotic genes were studied in glioma cells. The apoptosis rate of the U251 and LN229 cells in the CKS2‐siRNA group was significantly higher than that in the negative‐siRNA group, whereas the apoptosis percentage of U251‐CKS2 and LN229‐CKS2 cells at 48 h was lower than that of empty vector groups (*p* < 0.05, Figure 4A–D). The expression of pro‐apoptotic genes such as BAX and cleaved‐caspase3 was remarkedly upregulated in U251‐CKS2siRNA and LN229‐CKS2siRNA cells compared with those in control group. Meanwhile, the levels of anti‐apoptotic molecular BCL‐2 were down‐regulated significantly. The opposite trends were observed in glioma cells overexpressing CKS2 (*p* < 0.05, Figure 4E–H). Collectively, these findings revealed that CKS2 knockdown inhibited glioma proliferation and induced apoptosis in glioma cells and both pro‐apoptotic and anti‐apoptotic genes were involved.

### 3.4 CKS2 promotes glioma cell migration and invasive capability

**FIGURE 5:**
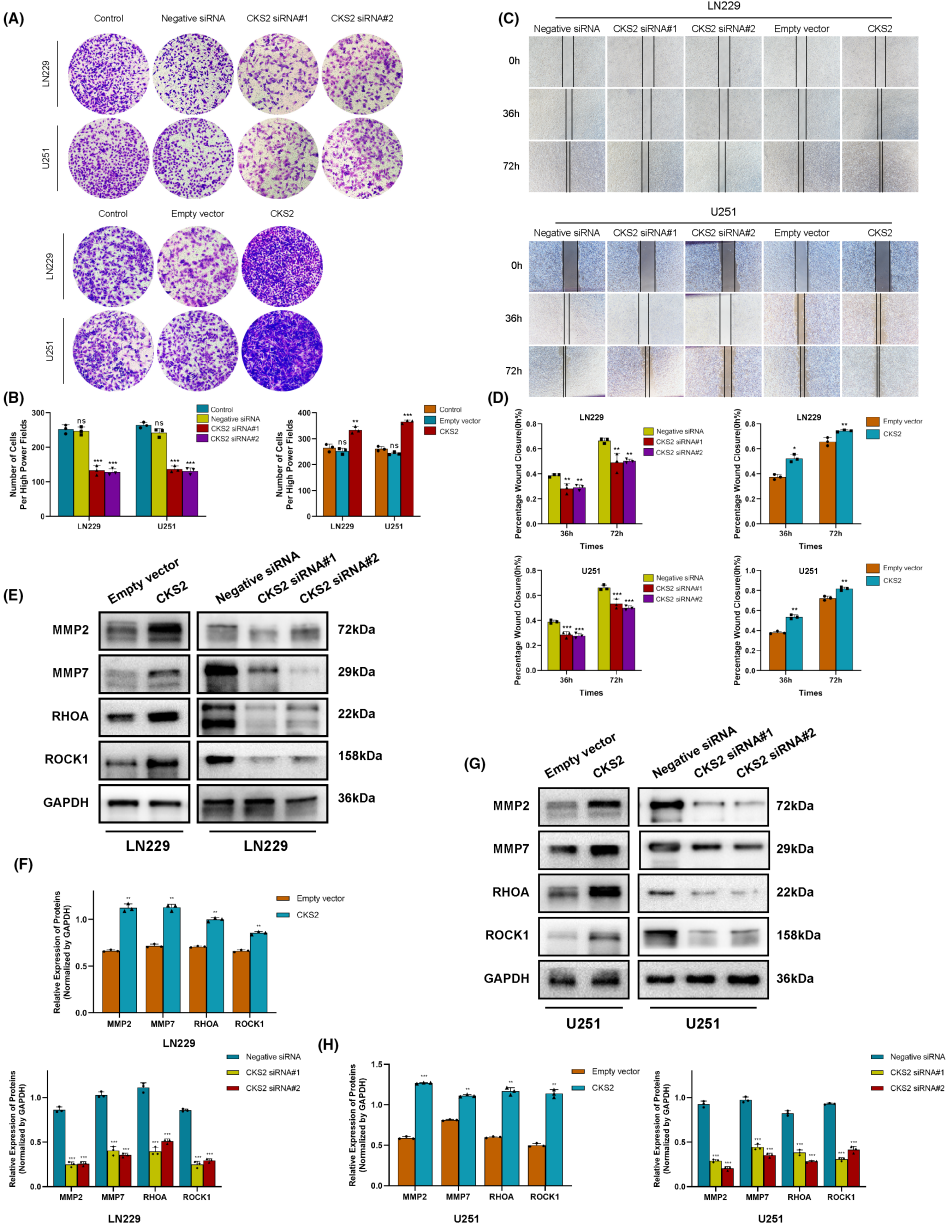
Effects of knockdown and overexpression of CKS2 on migration and invasion of glioma cells. (A, B) Transwell assay showed the change of invasive ability of U251 and LN229 cells after being transfected with negative siRNA, CKS2‐siRNA, empty vector, and CKS2 overexpression plasmid. (C, D) Wound‐healing assay of U251 and LN229 cells 0 h, 36 h, and 72 h after CKS2‐siRNA and overexpression plasmid transfection. (E, H) Western blot analysis to determine the expression changes of invasion markers in CKS2 overexpressing/silencing LN229 and U251cells. Quantitative graphs were shown in G and H.

To further understand the functions of CKS2 in gliomagenesis, we explore the effect of CKS2 on glioma cell migration and invasion. In Transwell assay, findings showed that glioma cells had attenuated invasion ability following transfection with CKS2‐siRNA, whereas the capability was increased after being transfected with CKS2 overexpression vector in U251 and LN229 (*p* < 0.05, Figure 5A–B). Consistently, glioma cells also exhibited enhanced migration capability in wound heal assay when treated with the CKS2 overexpression plasmid compared to the empty vector, while attenuated migration was observed following transfection with CKS2‐siRNA (*p* < 0.05, Figure 5C–D). Then, the effect of CKS2 expression on migration‐associated genes was examined by Western blot analysis. The results revealed that knockdown of CKS2 drastically down‐regulated the level of MMPs, RHOA, ROCK1 proteins in U251 and LN229 glioma cell lines compared to control group, while CKS2 overexpression markedly elevated the expression of aforementioned migration‐related genes in glioma cells compared to control (*p* < 0.05, Figure 5E–H). These data above indicated that CKS2 induced migration and invasion in glioma cells via regulating the expression of migration‐related proteins.

### 3.5 CKS2 promoted epithelial‐mesenchymal transition (EMT)‐like phenotypes in U251 and LN229 cell lines

**FIGURE 6:**
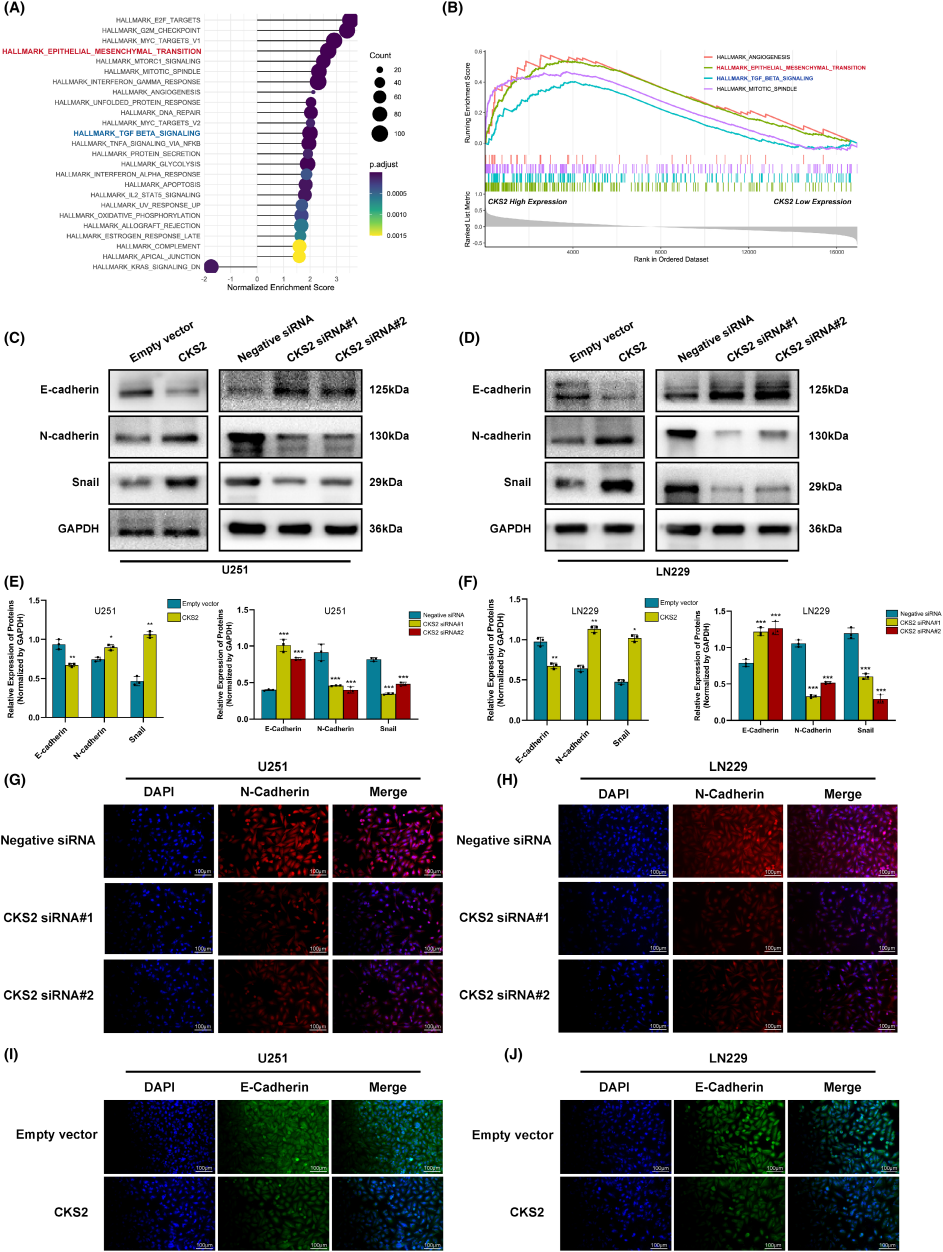
Inhibition of CKS2 reversed the epithelial‐mesenchymal transition. (A, B) Single‐gene related GSEA enrichment plots represented that CKS2‐associated enrichment of epithelial‐mesenchymal transition (EMT) pathway. (C–F) Western blotting showing the increased protein expression of epithelial marker (E‐cadherin) and the decreased levels of mesenchymal markers (N‐cadherin, Snail) after knockdown of CKS2. The reversed effect was observed after treatment with CKS2 overexpression plasmid. (G–J) Immunofluorescence staining showing the same outcomes as for U251 and LN229 cells after siRNA or expression plasmid treatment. The scale bar corresponds to 100 μm.

To provide further insights into the mechanisms of CKS2‐induced proliferation, we used GSEA to investigate the possible biological functional of CKS2 in glioma with GEO public dataset. GSEA results showed that CKS2 might be involved in epithelial‐mesenchymal transition‐like (EMT) process in glioma (Figure 6A, B). EMT‐like process is correlated with the enhanced activity of matrix metalloproteins (MMPs), which further induces glioma cell invasion via degrading extracellular matrix proteins. Unsurprisingly, we found that EMT‐related genes markedly influenced by CKS2 knockdown/overexpression through Western blotting analysis. The results indicated that the expression of mesenchymal markers (such as N‐cadherin and Snail) decreased with silencing CKS2, and the epithelial marker E‐cadherin gradually increased (*p* < 0.05, Figure 6C–F). Furthermore, we also confirmed the same effect on EMT‐like process markers with immunofluorescence staining experiment that demonstrated that the knockdown of CKS2 repressed both vimentin and N‐cadherin, whereas increase the expression of E‐cadherin in U251 and LN229 cells (*p* < 0.05, Figure 6G–J). Therefore, the results of Figure 4E also supported the evidence that CKS2 promoted EMT‐like malignant phenotype in gliomagenesis.

### 3.6 The TGFβ/SMAD signaling contributes to CKS2‐mediated glioma cell aggressiveness

**FIGURE 7:**
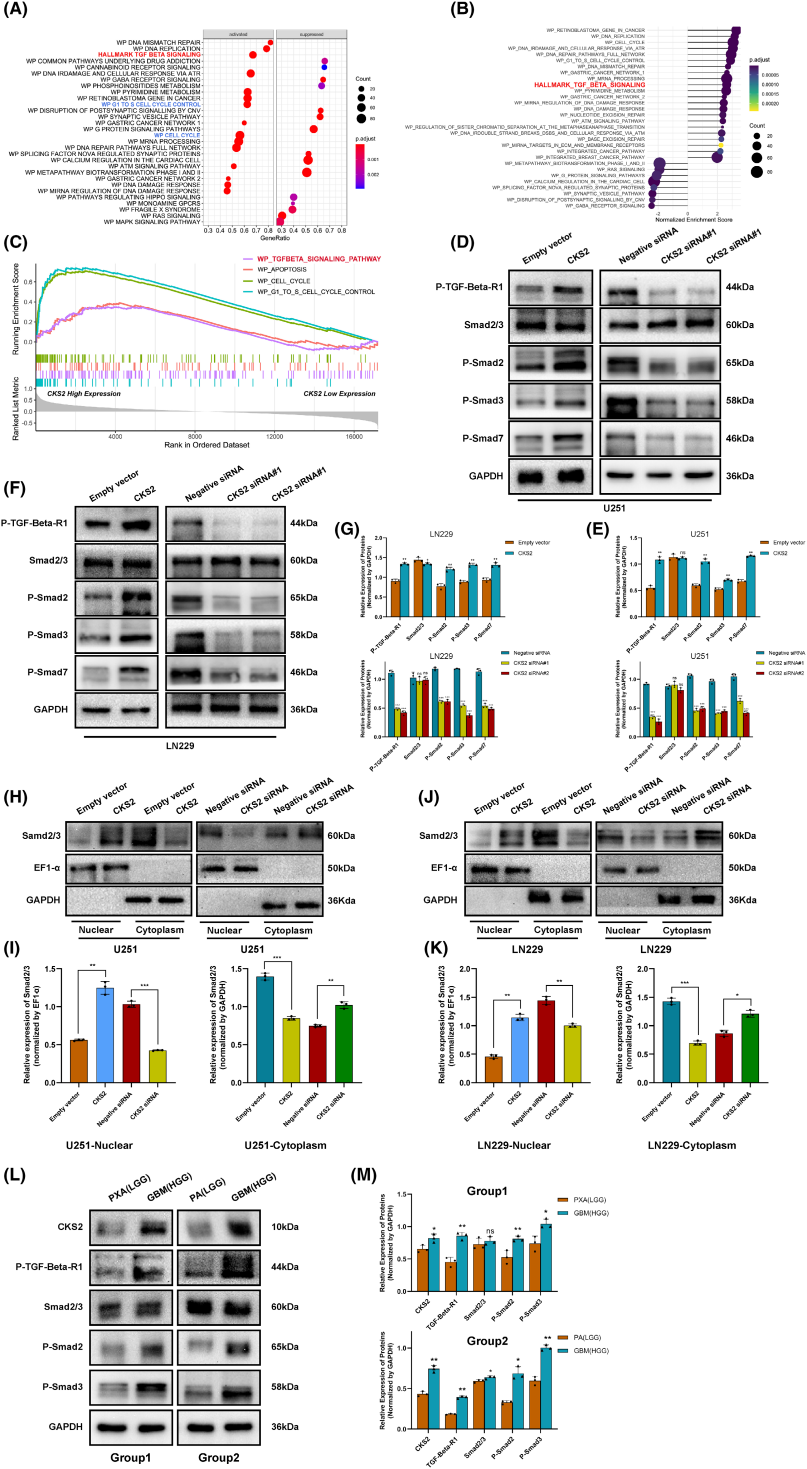
CKS2 promoted glioma cell aggressiveness by activating the TGFβ/SMAD signaling pathway. (A) The dot plot of partially enriched pathway. (B) The dot plot of the up‐ and down‐regulated pathways. (C) GSEA enrichment plot showed the upregulated pathway of TGFβ/SMAD. (D–G) Western blot detection of TGFβR1, smad2/3, P‐samd2, P‐smad3, P‐smad7 in U251 and LN229 cells CKS2‐siRNA or overexpression plasmid treatment. (H–K) Western blot detection of smad2/3 expression levels in cytoplasm and nucleus of U251 and LN229 cells treated with siRNA or overexpression plasmid. (L–M) Western blot assays detected the changes of TGFβ/SMAD pathway in low‐grade glioma (LGG) and high‐grade glioma (HGG) tissues.

To identify the major signaling pathway contributing to the CKS2‐induced oncogenic phenotype, CKS2‐associated GSEA enrichment analysis was performed. Firstly, the results of GSEA analysis identified 30 CKS2‐associated significantly enriched pathways (adj.*p*.Value < 0.05, Figure 7A, B), including 22 activated pathways (normalized enrichment score, NES >0) and 8 inactivated pathways (NES <0). As shown in Figure 7C, the TGFβ/SMAD signaling belonged to the activated signaling pathway, suggesting that CKS2 might promote gliomagenesis and activates the TGFβ signaling pathway. Thus, we confirmed the level of critical proteins in the TGFβ/SMAD pathway. As expected, knockdown of CKS2 significantly decreased but overexpression of CKS2 increased the level of downstream genes involved in TGFβ/SMAD signaling, such as P‐TGFβ1, P‐SMAD2, P‐SMAD3, and P‐SMAD7 (Figure 7D–G). Besides, the WB analysis also proved that the nucleocytoplasmic translocation of SMAD2/3 during the activation of CKS2‐mediated TGFβ/SMAD pathway (Figure 7H–K). Furthermore, to make our conclusion more convincing, we also verified the change of TGFβ/SMAD signaling in various types of glioma tissues (LGG and HGG) and normal brain (NB) tissues. Compared with low‐grade glioma tissues, high‐grade glioma with CKS2 overexpression showed obvious activation of TGFβ/SMAD signaling (Figure 7L–M).

**FIGURE 8:**
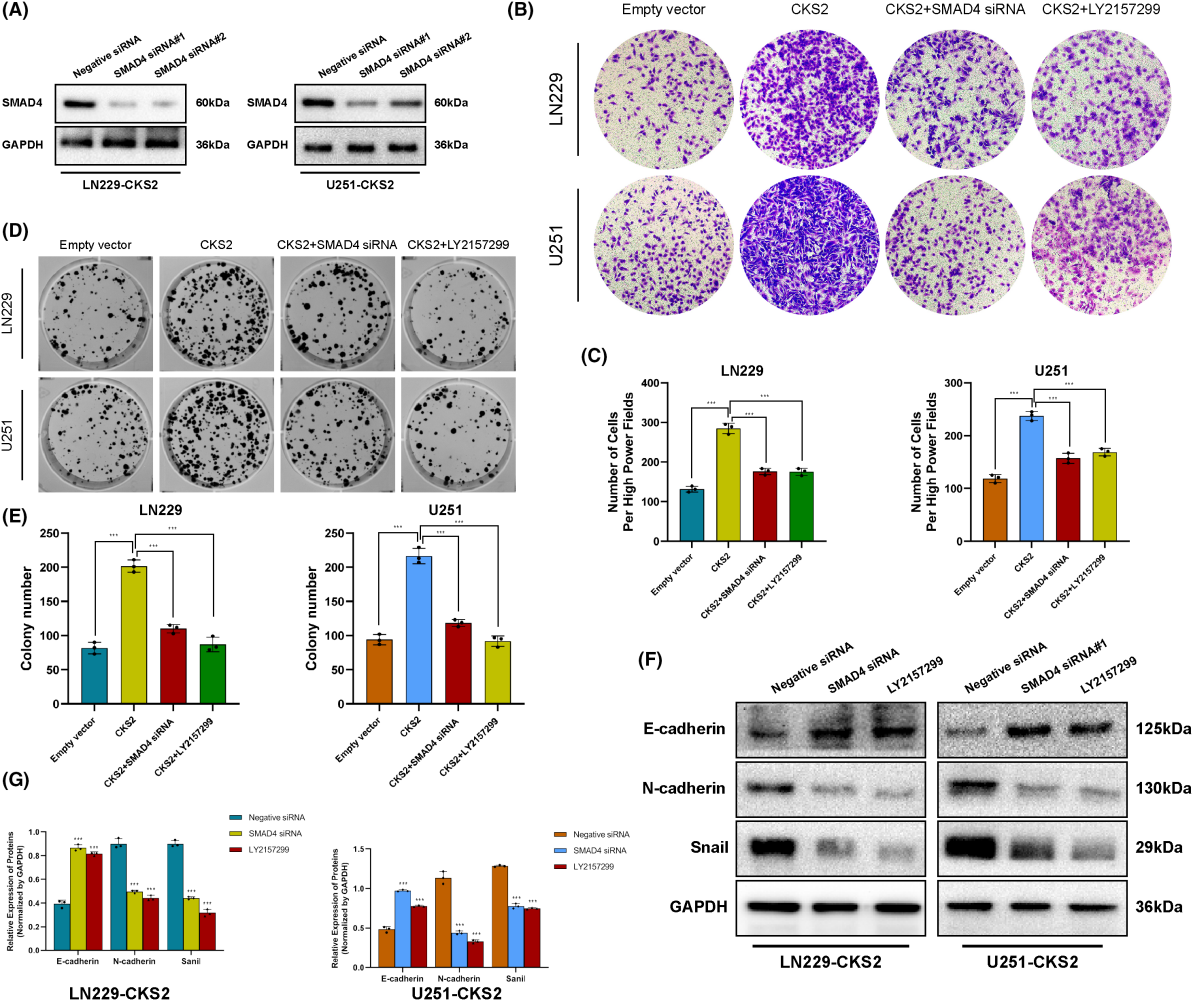
TGFβ/SMAD pathway contributes to CKS2‐mediated aggressiveness and malignant phenotype of glioma cells. (A) Western blotting analysis of SMAD4 expression in U251 and LN229 cell lines after siRNA treatment. (B, C) After being treated with SMAD4 siRNA or TGFβ inhibitor LY2157299, representative images of U251‐CKS2 plasmid and LN229‐CKS2‐plasmid cell invasion in the Transwell assay. (D, E) Representative micrographs of violet‐stained U251‐CKS2 and LN229‐CKS2 cell colonies treated with SMAD4 siRNA or LY2157299. (F, G) Western blotting analysis showed the protein level of EMT‐related markers (such as E‐cadherin, N‐cadherin, Snail) in LN229‐CKS2 and U251‐CKS2 after inhibiting TGFβ/SMAD pathway.

SMAD4, as the only co‐SMAD and central mediator of TGFβ/SMAD pathway, could bind to the promotor sequences of target genes and enhanced their transcription to active the signaling pathway. To further validate that CKS2‐induced gliomagenesis via activation of TGFβ/SMAD signaling, we block the TGFβ/SMAD pathway in CKS2 overexpression cells by transfecting the cells with SMAD4‐siRNA (Figure 8A) or TGFβ inhibitor LY2157299. As shown in Figure 8, inhibition of TGFβ/SMAD pathway by LY2157299 or SMAD4‐siRNA significantly attenuated the CKS2 overexpression‐mediated invasive capability (Figure 8B, C) and cell proliferation (Figure 8D, E) as well as EMT‐like process (Figure. 8F, G). These evidences mentioned above indicated that CKS2 induced malignant phenotype and tumorigenesis, at least in part, via activating TGFβ/SMAD signaling pathway.

## 4 DISCUSSION

Glioma is an enigmatic and invasive primary brain tumor with high recurrence and poor prognosis. On account of its disruption of the blood–brain barrier (BBB) and unlimited proliferation, it is dismal to effectively treat glioma by surgery, chemotherapy, and radio therapy.^2^, ^28^ Uncover the novel molecular mechanism of gliomagenesis is a crucial method to prevent tumor progress. In this study, we confirmed that a significant increase of CKS2 in glioma patients with CKS2 overexpression implied shorter overall survival time. Secondly, we verified that knockdown of CKS2 both sharply inhibited migration, invasion and induced apoptosis in glioma cells. Moreover, we found that CKS2 inhibition induced a series of changes in EMT markers expression, which indicated that CKS2 participated in the regulation of EMT‐like malignant phenotype in glioma. We also found that TGFβ/SMAD signaling pathway might regulate the CKS2‐drived EMT‐like process. Knocking down CKS2 effectively inhibit the activation of TGFβ/SMAD signaling pathway and dramatically decrease glioma proliferation; the phenomenon above was also observed in glioma tissues. Finally, we reported inhibition of TGFβ/SMAD pathway (by TGFβ inhibitor LY2157299 or SMAD4 siRNA) could reverse the tumor‐promoting effects caused by CKS2. Hence, we suggested that CKS2 promoted gliomagenesis and epithelial‐mesenchymal transition‐like process through TGFβ/SMAD signaling pathway.

Recently, CKS2 is widely accepted as a key tumor regulator, increasing number of researches have found that CKS2 gene copy number are increased in the various tumorigenesis and are correlated to the poor prognosis. Jonsson et al reported the major role of CKS2 in mitochondrial DNA replication in complex with SSBP1, which showed a novel link between cell proliferation by CKS2‐induced OXPHOS in the mitochondrion and chemoradioresistance of cervical cancer.^29^ In tongue squamous cell carcinoma, CKS2 promoted cell accumulation in G2/S phase and tumor progression via interacting with DUTPase and regulating its location in nucleus.^30^ Moreover, a research with the observation that CKS2 level was increased in higher histopathological grade breast cancer led to the suggestion that CKS2 might exert a pivotal role in tumor cell motility.^31^ Previous oligonucleotide microarray analysis also reported that CKS2mRNA was aberrantly elevated in glioma tissues; besides, CKS2 has been described as upregulation in high‐grade gliomas compared to the low‐grade glioma,^13^ however, whether CKS2 overexpression could stimulate glioma cell motility remained to be verified. In our study, we found that both center and border of glioma exhibited aberrantly elevated level of CKS2. Besides, we also found that glioma cells with CKS2 overexpression tended to influence adhesion with adjacent cells and change cellular biological behaviors, such as migration and invasion.

On the contrary, Zhong et al reported that LASP1 facilitated the EMT‐like process through upregulating Snail and activating PI3K/AKT signal pathway in LN229 and T98G glioma cell lines.^32^ Enhanced EMT transcription factor Slug was also observed in orthotopic glioma model mice, suggesting that checkpoint molecular B7‐H3 induce EMT‐like process and gliomagenesis.^33^ Lee et al illustrated that KITENIN maintained glioma stem cell markers (CD133, ALDH1, EPH‐B1) and accelerated migration and invasion by epithelial‐mesenchymal transition (EMT) process.^34^ Although there might be some different behavioral patterns between classical EMT process in epithelial tumor and the EMT‐like procession neuroepithelial tumors, the change of many EMT‐related biomarkers above played a crucial role in the gliomagenesis. Therefore, the term the “EMT‐like process” or “glial‐to‐mesenchymal transition” has been the focus in the tumorigenesis, especially in gliomagenesis.^35^, ^36^ Glioma, especially glioblastoma, is characterized by local infiltration and spread; however, neuroepithelial tumors have only been poorly investigated for extracranial metastases. Recently, Bang‐Christensen et al reported that recombinant VAR2CSA Malaria protein could be applied for capturing and detecting circulating tumor cells (CTCs) from both adult and pediatric glioma patients.^37^ Bednarz‐Knoll et al also illustrated that the epithelial‐mesenchymal transition (EMT) exerted an important role in forming metastases in the new tumor microenvironment after CTCs have settled down in distant organ.^38^ For examples, CTCs isolated from patients with metastastic breast cancer represented the environment of mesenchymal phenotype over tumor cells within primary carcinoma.^39^ Overall, MET‐like process might serve as a key molecular event that induced proliferation, invasion, and migration in glioma. According to our experimented data, we found that cyclin‐dependent kinase subunit2 (CKS2) promoted malignant phenotypes and EMT‐like process in glioma through TGFβ/SMAD signaling pathway; besides, many researches also supported our opinion. Recently, a vivo study also demonstrated that integrator complex 8 (INTS8) promoted hepatocellular carcinoma proliferation and invasion through EMT marker (E‐cadherin) upregulation and activation of TGFβ/SMAD signaling pathway.^40^ Saitoh et al reviewed that TGFβ/SMAD played a tumor‐promoting role in EMT‐associated responses, such as tumor proliferation, invasion, migration, and metastasis.^41^ Our experimental data and other studies above provide enough theoretical support and evidence for the molecular mechanism by which CKS2 induced EMT‐like process and tumorigenesis through activating TGFβ/SMAD signaling pathway in glioma.

In conclusion, our study reported that transcription factor CKS2 can promote glioma cell proliferation, invasion, and migration. Moreover, we primarily found that knockdown of CKS2 induced apoptosis and inhibited EMT‐like process. Finally, our data confirmed that CKS2 promoted malignancy and accelerated EMT‐like process by activating CKS2‐dependent TGFβ/SMAD signaling pathway. Taken together, cyclin‐dependent kinase subunit2 (CKS2) could serve as a novel target in the treatment of glioma.

## AUTHOR CONTRIBUTIONS

**Fan Feng:** Conceptualization (lead); data curation (lead); formal analysis (lead); investigation (lead); methodology (lead); visualization (lead); writing – original draft (lead). **Zongqing Zhao:** Methodology (equal). **Xuechang Cai:** Software (equal). **Xueyuan Heng:** Funding acquisition (lead); supervision (lead); writing – review and editing (lead). **Ximeng Ma:** Supervision (equal).

## FUNDING INFORMATION

The author(s) disclosed receipt of the following financial support for the research, authorship, and/or publication of this article: This work was supported by the Nature Science Foundation of Shandong Province (ZR2014HM077).

## CONFLICT OF INTEREST

No potential conflicts of interest were disclosed.

## ETHICS APPROVAL

The study protocol was approved by the Ethics Committees of Linyi People's Hospital (No.10023).

## INFORMED CONSENT

Written informed consent was obtained from all patients prior to sample collection.

## Supporting information


Table S1–S2

## Data Availability

The original contribution presented in the study is included in the article/supplement material. Future inquiries can be directed to the corresponding authors.
