# Supplementary material for: Cyclin‐dependent kinase subunit2 (CKS2) promotes malignant phenotypes and epithelial‐mesenchymal transition‐like process in glioma by activating TGFβ/SMAD signaling
Source: Cancer Med. 2022 Oct 25;12(5):5889–907. doi: 10.1002/cam4.5381 (PMC10028050; doi:10.1002/cam4.5381)
Supplement: Supplementary file 1 — Table S1–S2 [file CAM4-12-5889-s001.pdf]

Supplementary Table 1. Immunohistochemical analysis of CKS2 in glioma tissue

Group	Case	Score of CKS2 expression	
		Low (score < 4)	High (score > 4)
NB	8	8 (100.0)	0 (0.0)
Grade Ⅰ-Ⅱ	28	19(67.9)	9 (32.1)
Grade Ⅲ-Ⅳ	42	13 (31.0)	29 (69.0)

For Review Only

Supplementary Table 2. Clinical variables of patients in TCGA datasets

Characteristic	Low expression of CKS2	High expression of CKS2	p
n	348	348	
WHO grade, n (%)			< 0.001
G2	191 (30.1%)	33 (5.2%)	
G3	120 (18.9%)	123 (19.4%)	
G4	5 (0.8%)	163 (25.7%)	
IDH status, n (%)			< 0.001
WT	43 (6.3%)	203 (29.6%)	
Mut	302 (44%)	138 (20.1%)	
1p/19q codeletion, n (%)			< 0.001
codel	111 (16.1%)	60 (8.7%)	
non-codel	236 (34.3%)	282 (40.9%)	
Age, meidan (IQR)	39 (31, 50)	53 (39, 63)	< 0.001

view Only
